# Qualitative and Quantitative Analysis of Cardiac Progenitor Cells in Cases of Myocarditis and Cardiomyopathy

**DOI:** 10.3389/fgene.2018.00072

**Published:** 2018-03-06

**Authors:** Marie Gerisch, Jan Smettan, Sabine Ebert, Maria Athelogou, Beate Brand-Saberi, Nick Spindler, Wolf C. Mueller, Shibashish Giri, Augustinus Bader

**Affiliations:** ^1^Applied Stem Cell Biology and Cell Technology, Biomedical and Biotechnological Center, University of Leipzig, Leipzig, Germany; ^2^Division of Cardiology and Angiology, Department of Internal Medicine, Neurology and Dermatology, University Hospital Leipzig, Leipzig, Germany; ^3^Definiens AG, Munich, Germany; ^4^Department of Anatomy and Molecular Embryology, Institute of Anatomy, Faculty of Medicine, Ruhr-University Bochum, Bochum, Germany; ^5^Department of Orthopedics, Trauma and Plastic Surgery, University Hospital Leipzig, Leipzig, Germany; ^6^Department of Neuropathology, University Hospital Leipzig, Leipzig, Germany; ^7^Department of Plastic and Hand Surgery, University Hospital Rechts der Isar, Munich Technical University, Munich, Germany

**Keywords:** local cardiac stem/progenitor cells, c-Kit^+^ (CD117^+^) cells, CD90^+^ cells, myocarditis, cardiomyopathy, human myocardium biopsy

## Abstract

We aimed to identify and quantify CD117^+^ and CD90^+^ endogenous cardiac progenitor cells (CPC) in human healthy and diseased hearts. We hypothesize that these cells perform a locally acting, contributing function in overcoming medical conditions of the heart by endogenous means. Human myocardium biopsies were obtained from 23 patients with the following diagnoses: Dilatative cardiomyopathy (DCM), ischemic cardiomyopathy (ICM), myocarditis, and controls from healthy cardiac patients. High-resolution scanning microscopy of the whole slide enabled a computer-based immunohistochemical quantification of CD117 and CD90. Those signals were evaluated by Definiens Tissue Phenomics® Technology. Co-localization of CD117 and CD90 was determined by analyzing comparable serial sections. CD117^+^/CD90^+^ cardiac cells were detected in all biopsies. The highest expression of CD90 was revealed in the myocarditis group. CD117 was significantly higher in all patient groups, compared to healthy specimens (^*^p < 0.05). The highest co-expression was found in the myocarditis group (6.75 ± 3.25 CD90^+^CD117^+^ cells/mm^2^) followed by ICM (4 ± 1.89 cells/mm^2^), DCM (1.67 ± 0.58 cells/mm^2^), and healthy specimens (1 ± 0.43 cells/mm^2^). We conclude that the human heart comprises a fraction of local CD117^+^ and CD90^+^ cells. We hypothesize that these cells are part of local endogenous progenitor cells due to the co-expression of CD90 and CD117. With novel digital image analysis technologies, a quantification of the CD117 and CD90 signals is available. Our experiments reveal an increase of CD117 and CD90 in patients with myocarditis.

## Introduction

The World Health Organization reports cardiovascular diseases as the main cause of 29% of global death each year (Lozano et al., 2012). There are approximately 17.3 million cardiovascular-related deaths per year worldwide (Townsend et al., 2015). Particularly in the European region, cardiovascular diseases cause 4 million deaths; which is, 45% of death per year (Townsend et al., 2015). In all countries of Europe, the primary cause of death in women is cardiovascular diseases, estimated to be 51% of all-cause mortality in women. For men, 42% of deaths are caused by cardiovascular diseases (Townsend et al., 2015).

Due to the profound importance of cardiovascular diseases, the natural endogenous regenerative capacity of the human heart has been a topic of debate for decades. Accumulating evidence over the last decade has suggested that the human heart has the potential to undergo natural regeneration. Locally resident cardiac progenitor or stem cells might play a vital role toward the natural regeneration capacity of the heart. Myocyte proliferation happens to a low extent in the human heart, while enhanced proliferation was observed following injuries of the heart such as myocardial infarction (Beltrami et al., 2001). In addition, the muscle cells of the whole human heart are replaced every 4.5 years (Anversa et al., 2006). Cardiac progenitor cells (CPCs) can replicate in response to some pathological conditions, and they are also able to play an active role in the regeneration of injured parts of the heart (Gonzalez et al., 2008).

Of particular interest are cells with the c-Kit receptor (CD117 or SCFR-stem cell factor receptor) on the surface. Beltrami and colleagues reported the existence of CD117^+^ cells with characteristics of CPCs (Beltrami et al., 2003). In addition, other researchers described that cardiac CD117^+^ cells are potential local stem cells, which reside in the human heart (Castaldo et al., 2008; Di Meglio et al., 2010; Sandstedt et al., 2012). Bearzi and colleagues also described the typical stem cell features of the CD117^+^ cells: they are clonogenic, multipotent, and self-renewing (Bearzi et al., 2007). An increase in the number of cardiac CD117^+^ cells was observed in several cardiovascular diseases such as heart failure, cardiac hypertrophy, ischemic cardiomyopathy (ICM), acute cardiac injury, and pressure overload (Urbanek et al., 2003, 2005; Castaldo et al., 2008; Kubo et al., 2008; Altarche-Xifró et al., 2009; Itzhaki-Alfia et al., 2009; Rupp et al., 2012).

**Figure 1:**
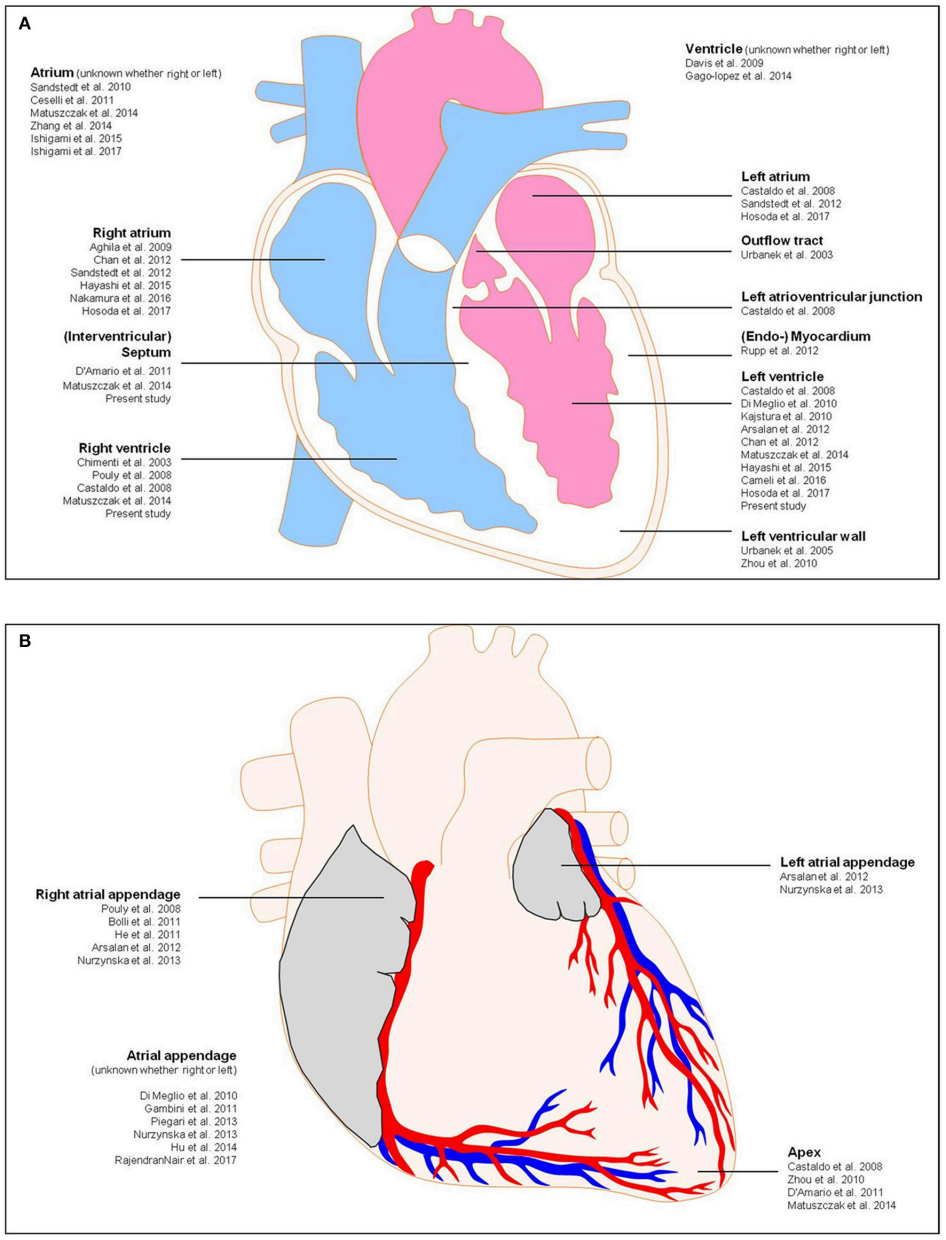
Schematic representation of the human heart showing biopsy locations used for previous identifications of CD117 and CD90. **(A)** Anterior view, **(B)** frontal section. Detailed information about the previous studies is shown in Table 1.

Nurzynska and colleagues conducted a comparative study of human CPCs in normal and pathological conditions (ischemic heart disease) and confirmed that the differentiation potential of CD117^+^ CPCs of the adult human pathological heart is weak in comparison to healthy cardiac tissue (Nurzynska et al., 2013). Bolli et al. conducted a phase I clinical trial for the clinical implication of CD117^+^ stem cells, and interestingly they found an increased cardiac functional capacity, reduced left ventricle scar size, and improved quality of life due to these cells (Bolli et al., 2011). They isolated CD117^+^ cells from 1 g myocardial tissue during cardiac surgery. They showed that the infusion of 1 million autologous CD117^+^ stem cells is not associated with noticeable adverse or significant positive effects. Therefore, the study of the anatomy of the human heart and the manner in which pathological states and micro-environmental conditions correlate with the availability of resident CPCs for cardiac tissue regeneration is required to further confirm the presence of local resident progenitor cells as well as for potential clinical strategies for novel forms of cardiac cell therapy by endogenous recruitment. Nevertheless, for progenitor cell detection, additional stem cell markers must be proven. Herein, we focus on the existence of CD90^+^ and CD117^+^ cells as regenerative precursor cells in the human healthy and diseased heart, that are responsible for the activation of endogenous resident progenitor cells toward tissue or cell injury. Previous investigations focused on the detection of CD90^+^ and CD117^+^ progenitor cells in the human heart along with additional information about biopsy location, diagnosis, patient age, and experimental structure are shown in Table 1. A schematic representation of the human heart provides an overview about biopsy locations used for previous identifications of CD90^+^ and CD117^+^ endogenous CPCs (Figure 1).

**Table 1:** Previous identifications of CD117 and CD90 in human along with patient age, diagnosis, and additional major details.

**Stem cell marker**	**Biopsy location**	**Diagnosis**	**Patient age in years**	**Culture/biopsy**	***In vivo/in vitro* expansion**	**Quantitative/qualitative analysis of CD117/CD90**	**Findings/comments**	**References**
CD117	Outflow tract	Aortic stenosis (*n* = 36) Control hearts (*n* = 12)	73 ± 10 71 ± 8	Formalin-fixed tissue	–	IHC	Increased number of stem-like cells in aortic stenosis	Urbanek et al., 2003
CD117	Right ventricle	DCM (*n* = 19) Idiopathic DCM (*n* = 10) Control hearts (*n* = 7)	73 ± 2 61 ± 4 76 ± 4	Tissue sections from biopsies with a size of nearly 3 mm^3^	–	IHC; Confocal microscopy	Cellular senescence and death of CD117^+^ cells leads to HF and premature cardiac aging	Chimenti et al., 2003
CD117	Left ventricular wall	Acute infarcts (*n* = 20) End-stage post-infarction CM (*n* = 20) Control hearts (*n* = 12)	62 ± 13 56 ± 7 60 ± 20	Formalin-fixed tissue	–	IHC	Increased number of CPCs in acute and chronic infarcts	Urbanek et al., 2005
CD117	Right ventricle; Right atrial appendage	Heart transplant recipients (*n* = 32) Chronic ICM (*n* = 18)	45.8 ± 11 65.3 ± 8.1	Formalin-fixed, paraffin-embedded tissue; Culture of right atrial appendage specimens	*In vitro*	IHC; Immuno-fluorescence; Confocal laser microscopy; Flow cytometry	Higher number of CD117^+^ cells in right ventricle than in atrial appendage; small number of CD117^+^ cells in cultured right atrial appendages	Pouly et al., 2008
CD117	Right + left ventricle; Left atrium; Left atrio-ventricular junction; Apex	End-stage HF with ICM (*n* = 20) Control hearts (*n* = 11)	55 ± 5.5 41 ± 12	Formalin-fixed, paraffin-embedded tissue; Isolation and culture from fragments of left ventricular myocardium	*In vitro*	Immuno-fluorescence	Increased number of CD117^+^ cells in ICM; Higher number of CD117^+^ cells in the atrial subepicardium than in the myocardium	Castaldo et al., 2008
CD117 CD90	Ventricle	Endomyocardial biopsy (*n* = 160) Heart transplant (*n* = 59) Unexplained CM (*n* = 12)	Recipients: 52 ± 14 Donors: 32 ± 12 CM: 49 ± 15	Direct culture and expansion of CPCs from myocardial tissue	*In vitro*	IHC; Confocal microscopy; Flow cytometry	Expansion and proliferation of CPCs is simple	Davis et al., 2009
CD117	Right atrium	Coronary artery disease (*n* = 30)	38–72	Culture of biopsy tissue, non-enzymatic isolation of CSCs	*In vitro*	Flow cytometry	Number of CSCs is not influenced by disease severity or risk factors for coronary artery disease	Aghila Rani et al., 2009
CD117	Left ventricular walls	Hearts from patients who died from non-cardiovascular diseases (*n* = 5)	<1–75	Formalin-fixed, paraffin-embedded tissue	–	IHC	A subpopulation of CD117^+^ cardiac cells may be authentic stem/progenitor cells	Zhou et al., 2010
CD117	Atrium	Coronary artery disease, Valvular disease, Atrial fibrillation (*n* = 43)	47–84	Directly isolated cells, monolayer and explant cultured cells	*In vitro*	Flow cytometry; RT-PCR	Number of CD117+ cells in directly isolated cells is lower than in monolayer culture	Sandstedt et al., 2010
CD117	Atrial appendage; Left ventricle	ICM (*n* = 20) Control hearts (*n* = 11)	55 ± 5.5 41 ± 12	Formalin-fixed, paraffin-embedded tissue; Epicardial cell culture from fragments of the appendages	*In vitro*	IHC; Immuno-fluorescence	Number of CD117^+^ cells increased in ICM, higher number in epicardium than in myocardium; EDCs partially express CD117	Di Meglio et al., 2010
CD117	Left ventricle	Hearts from patients who died from non-cardiovascular diseases (n = 74)	19–104	Formalin-fixed, paraffin-embedded tissue	–	ICC; Spectral Analysis	The female myocardium possesses more CSCs and younger myocytes than the male myocardium.	Kajstura et al., 2010
CD117 CD90	Atrium	ICM, Idiopathic CM, HCM, Valvular disease, Acromegaly (*n* = 23) Donor hearts (*n* = 18)	39–65 45.8 ± 15.7	Formalin-fixed, paraffin-embedded tissue; Isolation and expansion of CSCs	*In vitro*	Flow cytometry; Immunolabeling; RT-PCR; Spectral analysis	Number of CD117^+^ cells was higher in explanted hearts than in donor hearts	Cesselli et al., 2011
CD117	Right atrial appendage	Patients with postinfarction LV dysfunction, treated (*n* = 16) Controls (*n* = 7)	56 ± 8.8 57.3 ± 8.9	Isolation, expansion and intracoronary re-infusion of autologous CSCs	*In vivo*	Immunolabeling; Confocal microscopy; Flow cytometry	No adverse effects after infusion of CSCs; Improvement in left ventricular systolic function; Increased functional capacity; Reduced left ventricular scar size	Bolli et al., 2011
CD117	Right atrial appendage	During routine procedure (*n* = 30)	–	Fixed tissue sections, freshly isolated or cultured CSCs	*In vitro*	IHC; ICC; Flow cytometry; RT-PCR	Tissue sections and freshly isolated cells contain CD117	He et al., 2011
CD117 CD90	Atrial appendages	Patients undergoing aorto-coronary bypass grafting	–	Atrial appendage tissue specimens and cultured cells	*In vitro*	Immuno-fluorescence; Confocal analysis; Flow cytometry; qRT-PCR	CD117^+^ cardiac progenitors are primitive stem cells with multilineage differentiation potential; Possible relationship between CD117^+^cells and a heart-specific MSC population	Gambini et al., 2011
CD117 CD90	Right portion of the septum; Apex of the left ventricle	Patients undergoing cardiac transplantation LVAD implantation (*n* = 20)	23–67	Collection and expansion of CSCs	*In vitro*	Flow cytometry	Successful isolation and expansion to a clinically relevant number for autologous delivery	D'Amario et al., 2011
CD117	Left ventricle; Atrial appendages	Patients undergoing cardiac surgery	67 ± 2	Isolation of mononuclear cells; Analysis of formalin-fixed, paraffin-embedded tissue	–	Flow cytometry; IHC	Number of CSCs is higher in atria than in left ventricle	Arsalan et al., 2012
CD117	Endo-myocardium	Pressure overloaded single right ventricles (*n* = 8) DCM (*n* = 4) Heart transplant (*n* = 14)	<1–19	Formalin-fixed, paraffin-embedded tissue;	–	IHC; Confocal microscopy	Number of CD117^+^ cells is increased in human hearts exposed to pressure overload	Rupp et al., 2012
CD117	Right and left atrium	Patients undergoing cardiac surgery (*n* = 17)	32–79	Isolation and differentiation of side population cells	*In vitro*	Flow cytometry; RT-PCR	Identification of side population cells in left atrial biopsies	Sandstedt et al., 2012
CD117 CD90	Right atrium; Left ventricular epicardium	Chronic IHD (*n* = 22)	67 ± 2	Isolation and culture of explant- and CDCs	*In vitro*	Flow cytometry; Immuno-fluorescence	No routine culture of CDCs from ventricular epicardial biopsies; atrial and ventricular epicardial CDCs comprise few CD117^+^ cells & a various number of CD90^+^ cells	Chan et al., 2012
CD117	Atrial appendages	Oncologic patients with CHF (*n* = 6) and without CHF (*n* = 2) Control hearts (*n* = 6)	53 ± 6 63, 61 50 ± 9	Isolation and culture of CPCs and treatment with doxorubicin	*In vitro*	Immuno-fluorescence	Doxorubicin exposure adversely affects the population of CPCs and their function	Piegari et al., 2013
CD117	Atrial appendage	Patients with end-stage HF due to ICM undergoing heart transplants (*n* = 9) Control hearts (*n* = 9)	55.8 ± 3.1 50.4 ± 4.1	Isolation and proliferation of CD117^+^ cells	*In vitro*	Immuno-fluorescence	CD117^+^ cells do not reach terminal differentiation and functional competence in pathological conditions	Nurzynska et al., 2013
CD117	Atrium	Patients undergoing CABG surgery (*n* = 3)	52–65	Isolation and expansion of CSCs	*In vitro*	ICC; Flow cytometry	Characterization of ion-channels in CD117^+^ cells from all patients	Zhang et al., 2014
CD117 CD90	Ventricle	DCM; ICM; CHD (*n* = 32)	<1–59	Enzymatic processing of heart tissue, Culture and differentiation of cardiospheres	*In vitro*	Immuno-fluorescence; Confocal microscopy; Flow Cytometry; RT-PCR	CD117^+^ cells also expressed CD34, CD90, CD31, or CD144; CD90^+^ cells expressed mesenchymal cell markers and showed incomplete differentiation into cardiomyocyte—like cells	Gago-Lopez et al., 2014
CD117 CD90	Right and left ventricle; Intra-ventricular septum, Atrium, Apex	Explanted hearts removed during heart transplant surgery, including IHD, DCM, HCM, congenital heart defect (*n* = 26)	3–65	Formalin-fixed, paraffin-embedded tissue; Isolation and primary cardiac cell culture from tissue fragments	*In vitro*	IHC; Flow cytometry	Identification of CD117^+^ cells directly in myocardial tissue and CD117^+^ and CD90^+^ cells in cell culture	Matuszczak et al., 2014
CD117	Appendages	Patients undergoing cardiac surgery (*n* = 105)	1–78 (55.6 ± 17.0)	Isolation and culture of CSCs	*In vitro*	Flow cytometry	The percentage of CD117^+^ CSCs decreases with age, DM and CHD	Hu et al., 2014
CD117	Right atrium; Left ventricle	Patients undergoing left ventriculoplasty due to ICM (*n* = 10)	65.1 ± 9.1	CSC isolation and culture	*In vitro*	ICC; Fluorescent microscopy	Successful preparation of CD117^+^ CSCs	Hayashi, 2015
CD90	Atrium	Hypoplastic left heart syndrome (*n* = 14)	1.8 ± 1.5	Isolation and expansion of autologous CDCs followed by intracoronary infusion	*In vivo*	Flow cytometry	Intracoronary infusion of autologous CDCs is safe and practicable	Ishigami et al., 2015
CD117	Left ventricle	ICM and end-stage HF submitted to LVAD implantation (*n* = 4)	–	Formalin-fixed, paraffin-embedded tissue	–	IHC	CSCs are present in left ventricular apical segment of patients with LVAD implantation	Cameli et al., 2016
CD90	Right atrium	Patients who underwent heart surgery (*n* = 26)	2–83	Isolation and culture of CDCs	*In vitro*	Flow cytometry	Age has a limited influence on the quantity and quality of CDCs	Nakamura et al., 2016
CD117 CD90	Atrial appendage	Patients who underwent CABG surgery	–	Isolation, culture as CDCs, simulation of HR injury	*In vitro*	Flow cytometry	CDCs showed expression of CD117 and CD90, CDCs have greater resistance to HR injury compared to MSCs	RajendranNair et al., 2017
CD117	Right atrium; Left atrium; Left ventricle	Valvular heart diseases (*n* = 8); Patients receiving left ventriculoplasty (*n* = 13)	66.1 ± 10.0	Isolation and culture of CSCs from fresh and frozen tissue	*In vitro*	ICC	Cryopreservation has no influence on proliferative potential of CSCs	Hosoda et al., 2017
(Ishigami et al., 2015)CD90	Atrium	Single ventricle physiology (*n* = 41)	≤20	Isolation, expansion and intracoronary infusion of autologous CDCs	*In vivo*	Flow cytometry	Intracoronary infusion of CDCs improved cardiac function	Ishigami et al., 2017
CD117 CD90	Right ventricle; Left ventricle; Septum	DCM (*n* = 7) ICM (*n* = 10) Myocarditis (*n* = 3) Control hearts (*n* = 3)	DCM: 44 ICM: 58 Myocarditis: 24 Control hearts: 35 (mean values)	Formalin-fixed, paraffin-embedded tissue	–	IHC; Digital image analysis	Identification of CD117^+^ and CD90^+^ cells directly in myocardial tissue, CD117 is increased in ICM, DCM and myocarditis in comparison to control hearts	Present study

*CABG, Coronary artery bypass graft; CDC, cardiosphere-derived cell; CHD, Coronary Heart Disease; CHF, congestive heart failure; CM, cardiomyopathy; CPC, cardiac progenitor cell; CSC, cardiac stem cell; DCM, dilatative cardiomyopathy; DM, Diabetes Mellitus; EDC, Epicardially-derived cell; HCM, hypertrophic cardiomyopathy; HF, heart failure; HR, hypoxia–reoxygenation; ICC, Immunocytochemistry; ICM, ischemic cardiomyopathy; IHC, Immunohistochemistry; IHD, ischemic heart disease; LVAD, Left ventricular assist device; MSC, mesenchymal stem cell; RT- PCR, real time polymerase chain reaction*.

In the current study, we obtained myocardium biopsies from 23 patients with the following diagnoses: dilatative cardiomyopathy (DCM), ICM, myocarditis, and controls from cardiac healthy. The collected material was characterized by immunohistochemistry. Currently, paraffin-based histopathological tissue analysis represents the main conventional method for confirmation of presence or absence of histological markers, grading, or the quantification of stem cells markers in ready tissue sections. Additional quantification of these histopathological slides using an automated image analysis perspective, though providing with more sensitive and qualitative information on the presence of local CPCs in myocardium biopsies, represents a new set of challenges. In our previous studies, we compared immunohistochemical data with an automated image analysis method of digitized slides by Definiens Tissue Studio software (Abraham et al., 2005; Kaemmerer et al., 2014; Neubauer et al., 2016). In several cases, we quantified tissue morphology, staining distribution and intensity of staining, using both automated image analysis and manually performed slides (Abraham et al., 2005; Kaemmerer et al., 2014; Neubauer et al., 2016). Interestingly, in this image analysis, the digital image processing is performed by digitized histological slides, which results in numerous advantages over the conventional immunohistochemical method.

In the present study, we combined a fully automated digital image analysis with conventional histological slides to more sensitively confirm the presence of potential local endogenous CPCs and to perform a quantitative analysis of the cardiac cell signals in human myocardium biopsies from patients with various cardiac diseases.

## Materials and methods

### Patients and tissue samples

Ready sections (*n* = 69) of paraffin-embedded human endomyocardial biopsies from 23 different patients were generously provided after the patient's consent by Prof. K. Klingel (Department of Molecular Pathology, University of Tuebingen, Germany). These biopsies were obtained from cardiac healthy subjects (*n* = 3), patients with myocarditis (*n* = 3), DCM (*n* = 7), or ICM (*n* = 10). The biopsies were derived from the right as well as from the left ventricular myocardium and septum. The mean age of the patients with DCM was 44 years, the average duration of illness amounted 18 years. Patients with ICM had a mean age of 58 years and an average duration of illness of 10 years. In addition, patients with myocarditis had a mean age of 24 years with duration of illness of 1–6 months. Control patients had a mean age of 35 years. Human skin tissue (University of Leipzig, Department of Orthopedics, Trauma and Plastic Surgery) was used as positive control, negative control, and IgG–control. The investigations were approved by the local ethics committee (050-2010-08032010) and conducted in accordance with the principles of the Declaration of Helsinki World Medical Association (1975). Control tissues also included human cerebellum (University of Leipzig, Neuropathology Department) and kidney (University of Leipzig, Institute of Pathology).

### Immunohistochemistry

Two serial sections of each patient were utilized, one for CD90 staining and the other for CD117 staining, and a partial third one for CD105. The skin specimens were then embedded. The paraffin sections, 8–10 μm in thickness were cut with a rotary microtome (model RM2165; Leica Microsystems). The slides were deparaffinized and rehydrated. Afterwards, cells were blocked with 0.6% H_2_O_2_ in phosphate-buffered saline (PBS; pH 7.4) and washed in PBS/0.3% Triton-x. For antigen retrieval, the heat-induced epitope retrieval method was used. The slides were incubated in retrieval solution (10 mM citrate buffer) in a water bath set to 60°C overnight. On day 2, the slides were washed in PBS and blocked for 30 min at room temperature with 5% normal goat serum in PBS. Primary antibodies, in specific, CD90 [anti-CD90/Thy-1 antibody, rabbit monoclonal IgG, clone EPR3133, ab 133350 (dilution 1:100) Abcam Cambridge, UK] and CD117 [anti-c-kit/CD117 antibody, rabbit monoclonal IgG, clone YR145, ab 32363 (dilution 1:50) Abcam Cambridge, UK] were added and incubated overnight at 4°C. All primary antibodies are particularly suitable for immunohistochemistry of paraffin sections (IHC-P). For supplementary investigations, a further primary antibody was applied on an additional serial section [anti-CD105 antibody, rabbit monoclonal IgG, clone EPR10145, ab 169545 (dilution 1:200) Abcam Cambridge, UK]. Negative control staining was performed whereby the primary antibody was omitted. As isotype control served rabbit monoclonal IgG [clone EPR25A, ab172730 (dilution 1:100) Abcam Cambridge, UK]. The images of the positive and negative controls can be found in the Supplementary Material.

On day 3, the slides were washed in PBS and incubated with the secondary antibody [secondary horseradish peroxidase conjugated goat anti-rabbit IgG (H + L), 111-035-003 (dilution 1:100 in PBS + 1% goat serum + 1% human serum), Dianova Jackson Immuno-research Hamburg, Germany], for 45 min at room temperature, followed by incubation with 3-amino-9-ethyl-carbazole (AEC) in sodium acetate buffer (0.1 mol/L, pH 5.2) containing hydrogen peroxide. After rinsing, the sections were counterstained with hematoxylin Lillie's modification (ready-to-use formulation; DakoCytomation, Copenhagen, Denmark) and mounted in glycergel (Kaiser's glycerol gelatine; Merck KGaA, Darmstadt, Germany).

### Image analysis

Scanning of the complete slide was performed by Virtual Microscope Olympus VS 120 (Ruhr-University Bochum Faculty of Medicine, Anatomy and Molecular Embryology). Individual image processing and optimization were performed via cellSens Software (OLYMPUS Germany). Fully automatic image analysis using the Definiens Tissue Phenomics® Technology (DEFINIENS AG, Munich, Germany) and the image analysis platform Developer XD enabled a quantitative image analysis of the whole slide. The analysis was made with the original image files (^*^vsi), which were created by the Olympus Virtual Slide Microscope using the 20x objective and have a very high resolution until the cellular level. A tailored image analysis solution (rule set) was developed using Definiens AG Software. The rule set separated first foreground (tissue regions) from the background (image regions without tissue) and excluded tissue along the edge to avoid artifacts. In the next step, the solution segmented and classified signals according to their individual morphology and their relative staining intensity. Afterwards, the solution reclassified the signals into two groups: in single signals and concatenated signals. The relative number of CD90^+^ signals (total number of signals divided by the total area of tissue section), the relative area of CD90^+^ signals and relative area of CD117^+^ signals (total area of signals divided by the total area of tissue section) were calculated.

To classify the cells as local progenitor cells, the detection of several stem cell markers was necessary. As we utilized only one antibody per slide, the identification of cells with co-expression of several markers was done through visual comparison of two or rather three serial sections each. A representative field (4 mm^2^) with high histological quality was chosen to account for the variable size of the tissue samples. The total number of cells, which expressed both stem cell markers, CD90 and CD117, was counted and normalized per square millimeter. This procedure was performed in three examples of each group; the ones that had the best histological quality were chosen.

### Statistics

The data obtained from digital image analysis are best analyzed on the logit scale [logit (*p*) = log (*p*/(1 − *p*))]. Analyses were performed in Microsoft Excel 2007. The statistical significance of differences between groups was evaluated by ANOVA (analysis of variance, one way), followed up by a *post-hoc* test (Tukey-Kramer method). *P* < 0.05 was considered statistically significant. The results from co-expression analysis were expressed as the mean ± standard deviation.

## Results

### Identification of CD90^+^ and CD117^+^ cells

**Figure 2–10:**
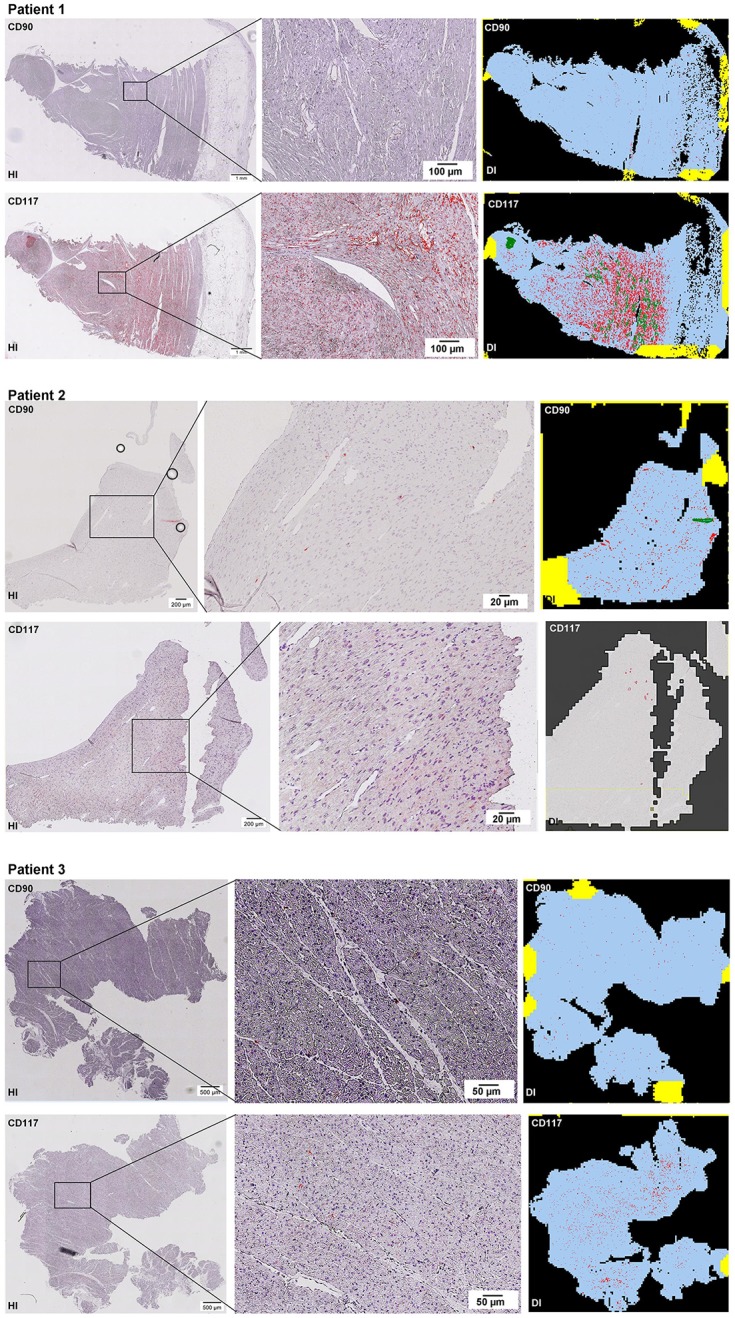
Histological and digital images of all tissue sections (*n* = 92). *(HI)* Histological whole slide images scanned with virtual slide microscope VS120 and *(DI)* reprocessed images, resulting from digital image analysis using the Definiens Tissue Phenomics® Technology. **(2)** Healthy cardiac patients, **(3)** patients with Myocarditis, **(4-7)** patients with ICM, **(8-10)** patients with DCM. Full-size images are available for download (https://figshare.com/s/13e838d63dfeac772894).

**Figure 3:**
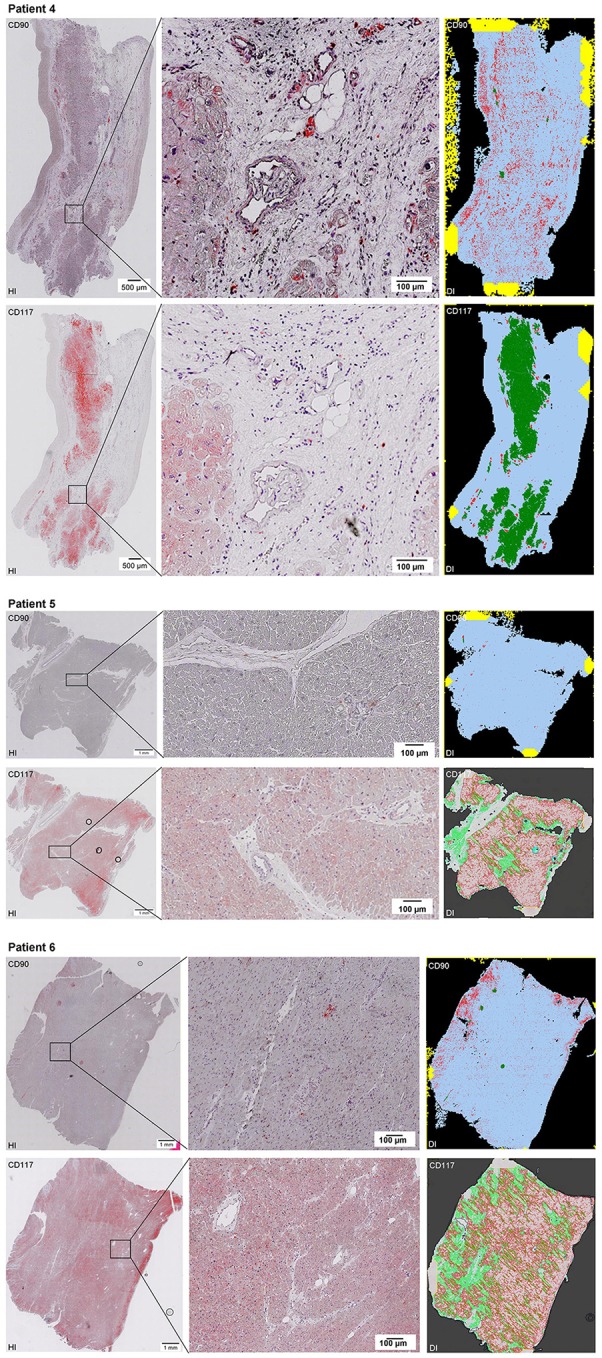
See Figure 2.

**Figure 4:**
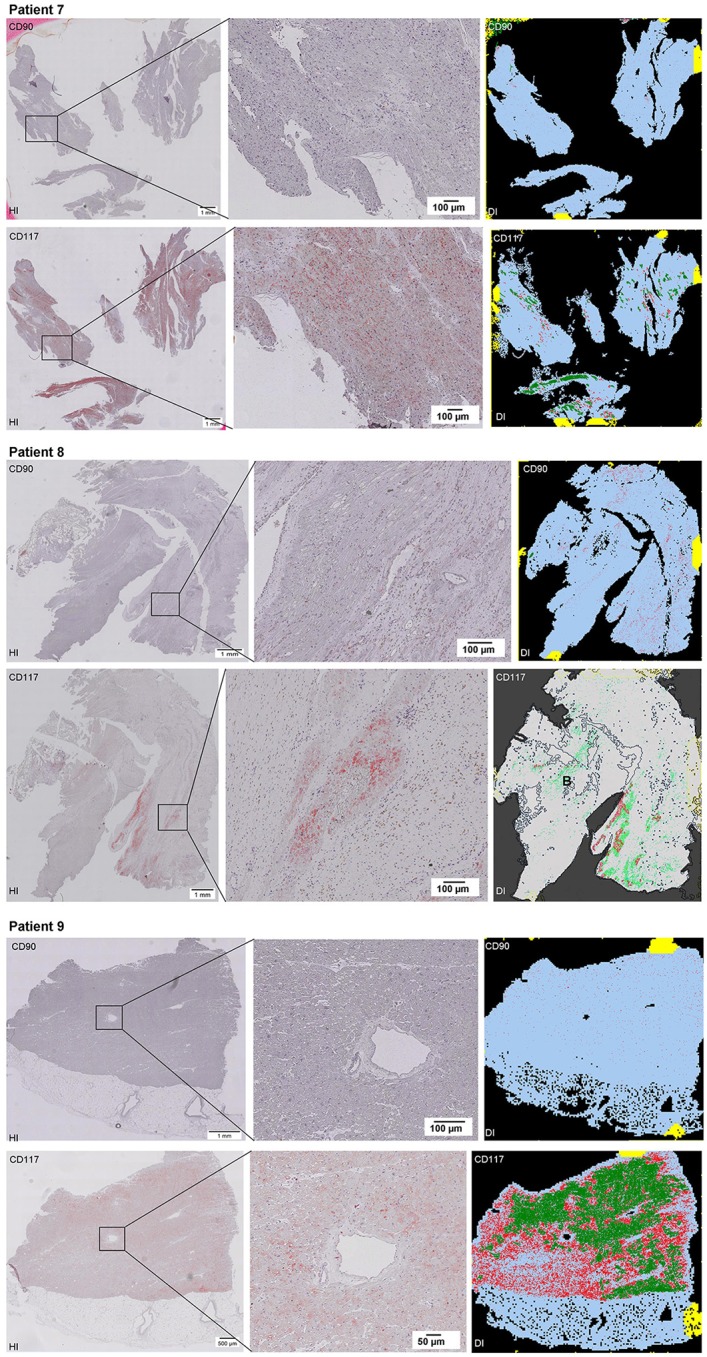
See Figure 2.

**Figure 5:**
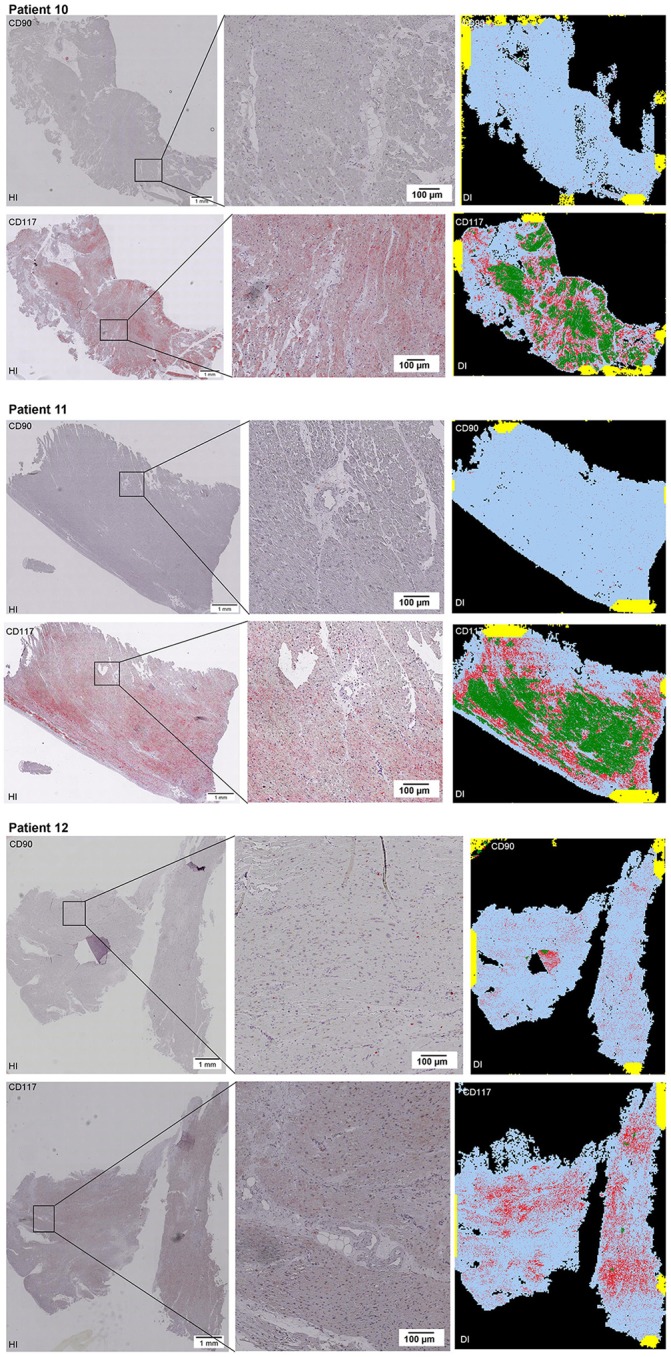
See Figure 2.

**Figure 6:**
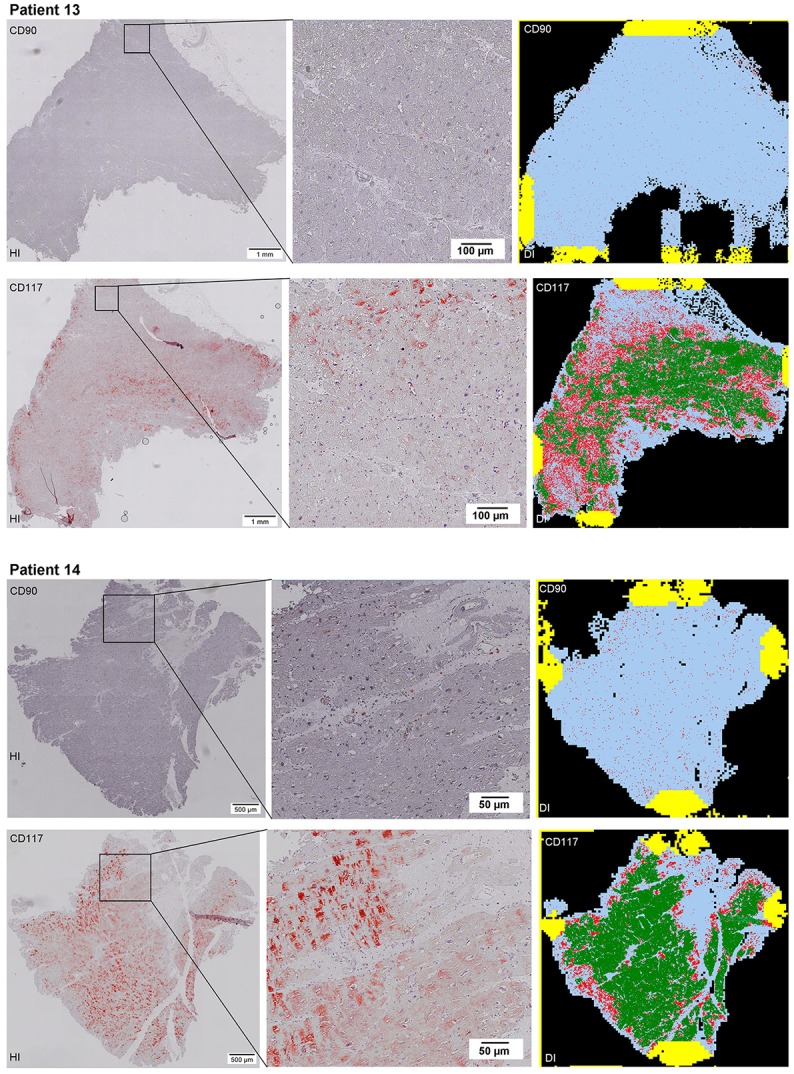
See Figure 2.

**Figure 7:**
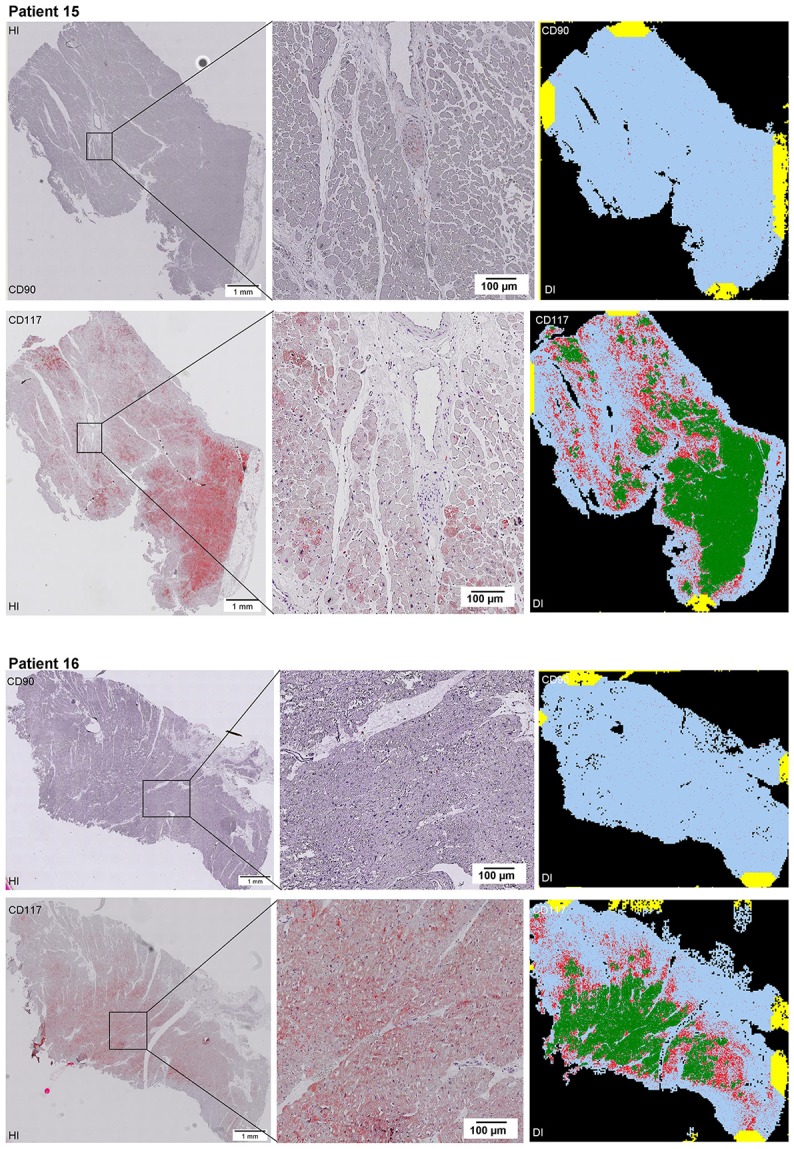
See Figure 2.

**Figure 8:**
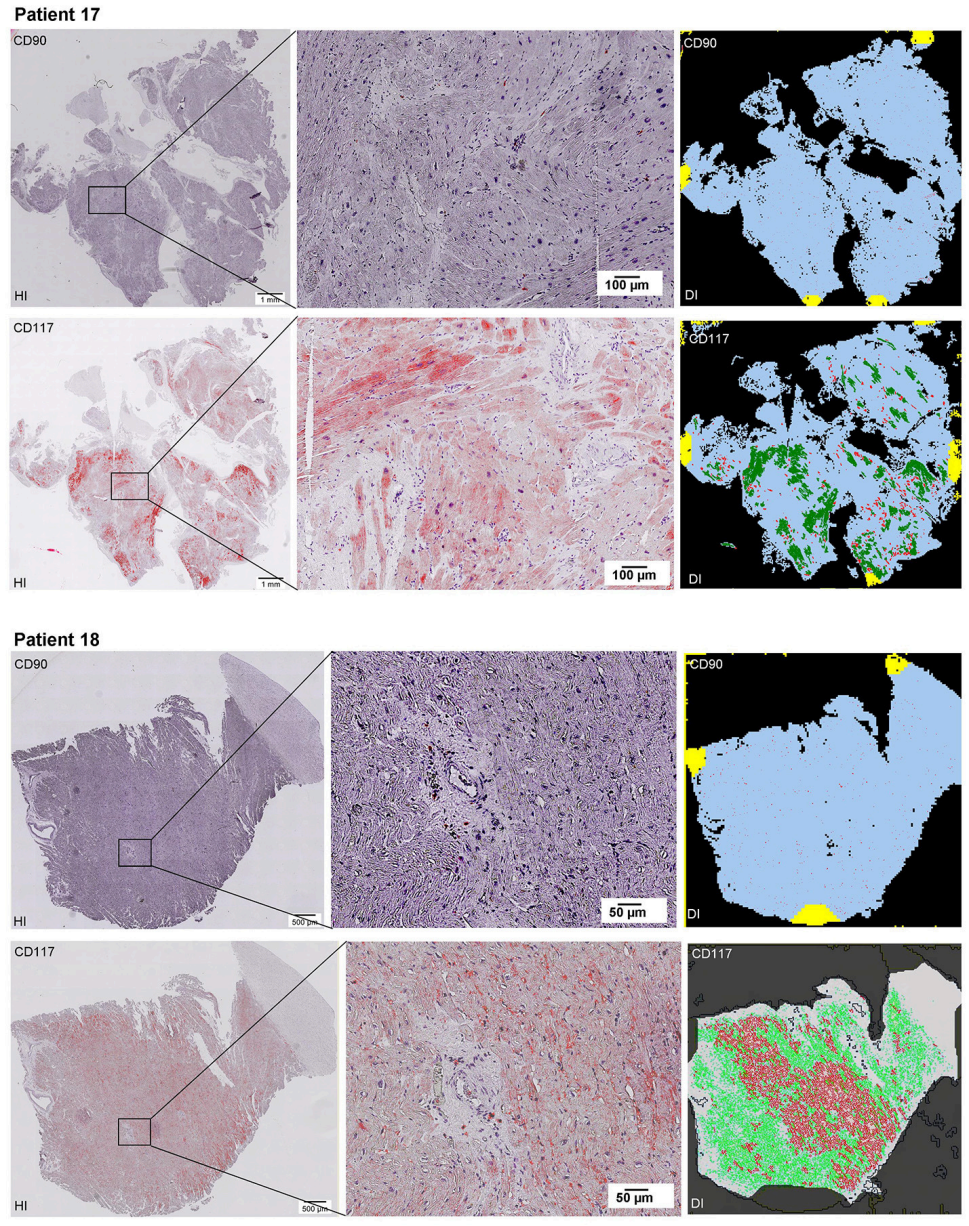
See Figure 2.

**Figure 9:**
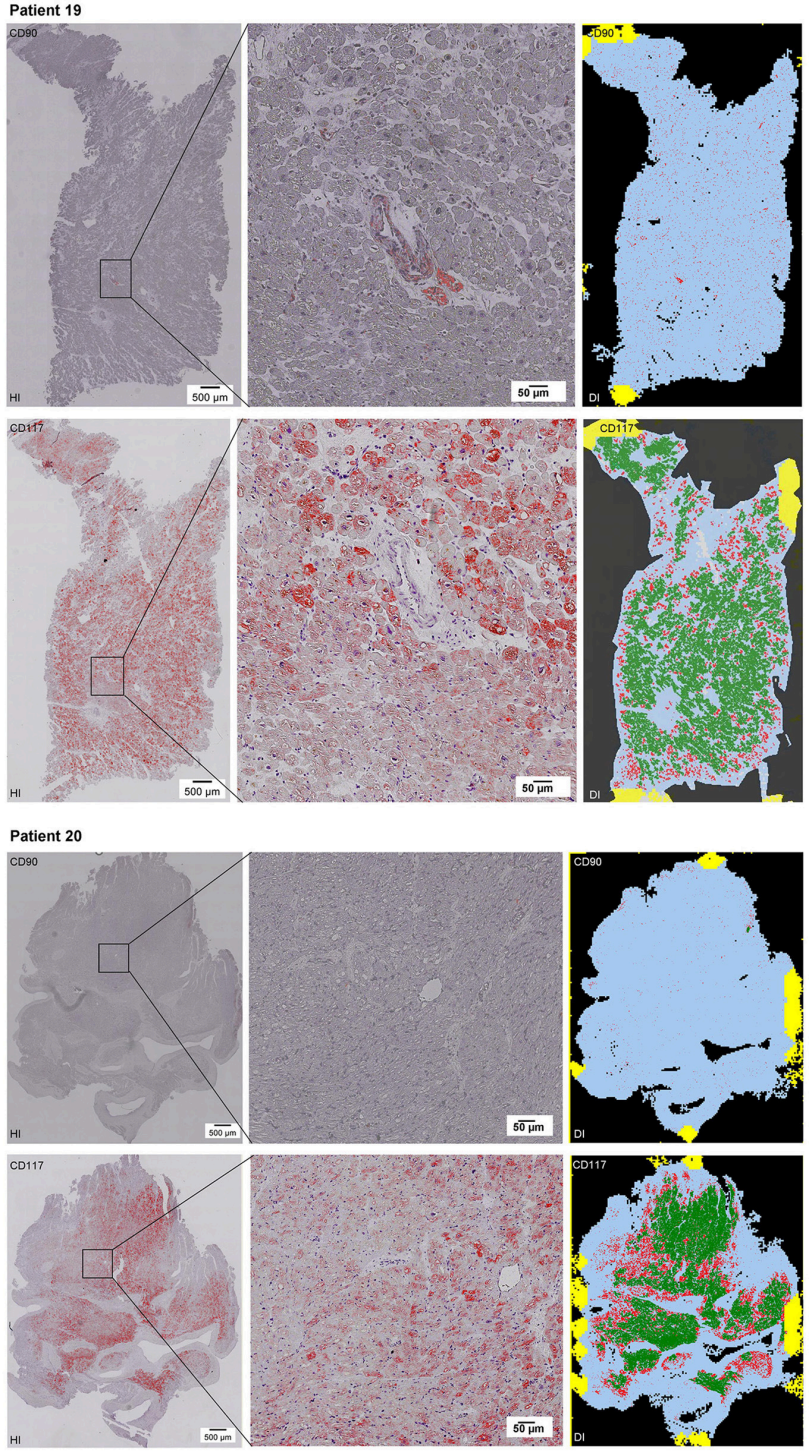
See Figure 2.

**Figure 10:**
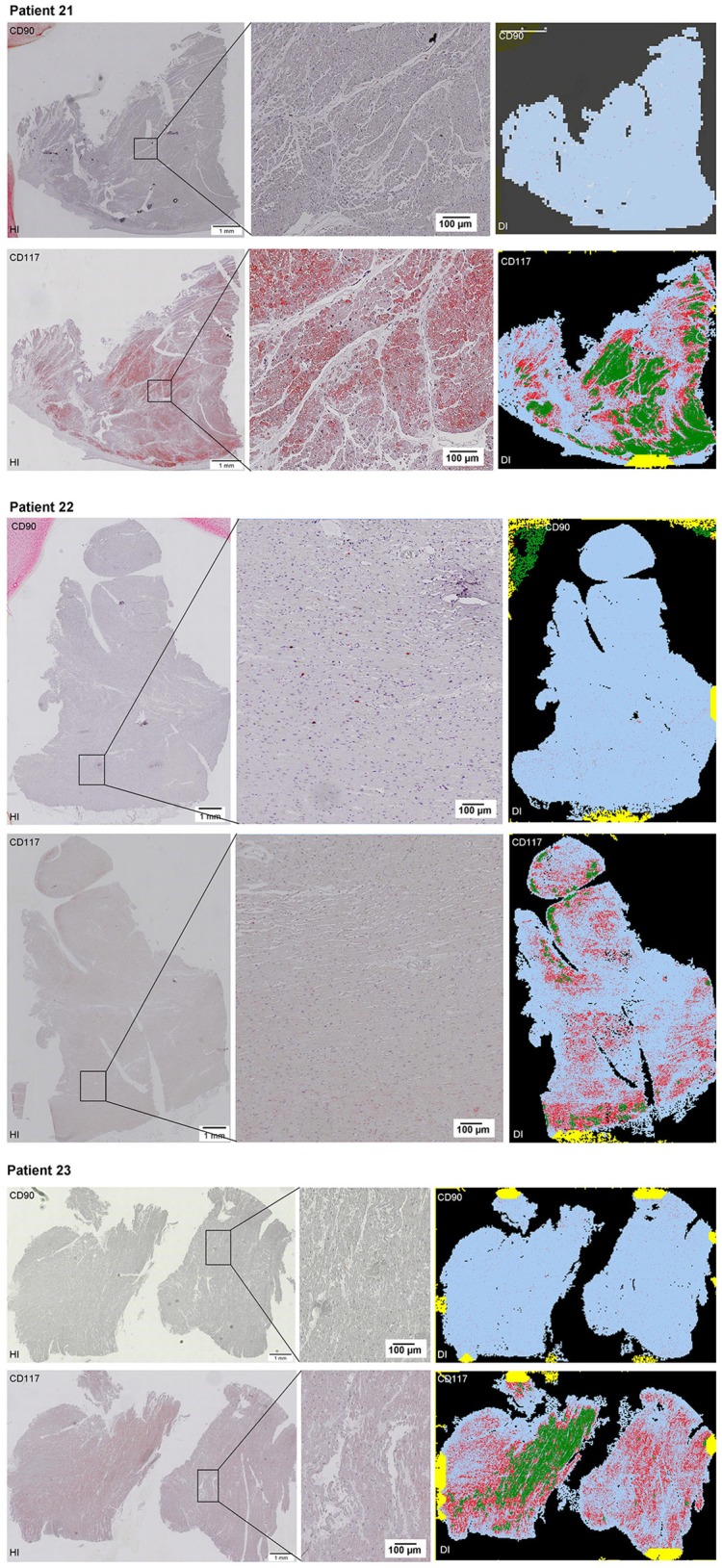
See Figure 2.

Scanning of the whole slide enabled a detailed view of the tissue sections in entirety and provided the basis for creating new images for the digital image analysis (Figures 2–10; high resolution images are available for download here: https://figshare.com/s/13e838d63dfeac772894). The histological analysis showed positive CD90 and CD117 signals in every group. CD90^+^ cells were detected as individual signals between the cardiomyocytes. In contrast, the CD117^+^ signals were identified both in cardiomyocytes and in cells which are located between the muscle cells. In many patients, the CD117 staining of the cardiomyocytes seems to predominate and only few signals in the interstitium were proved.

**Figure 11:**
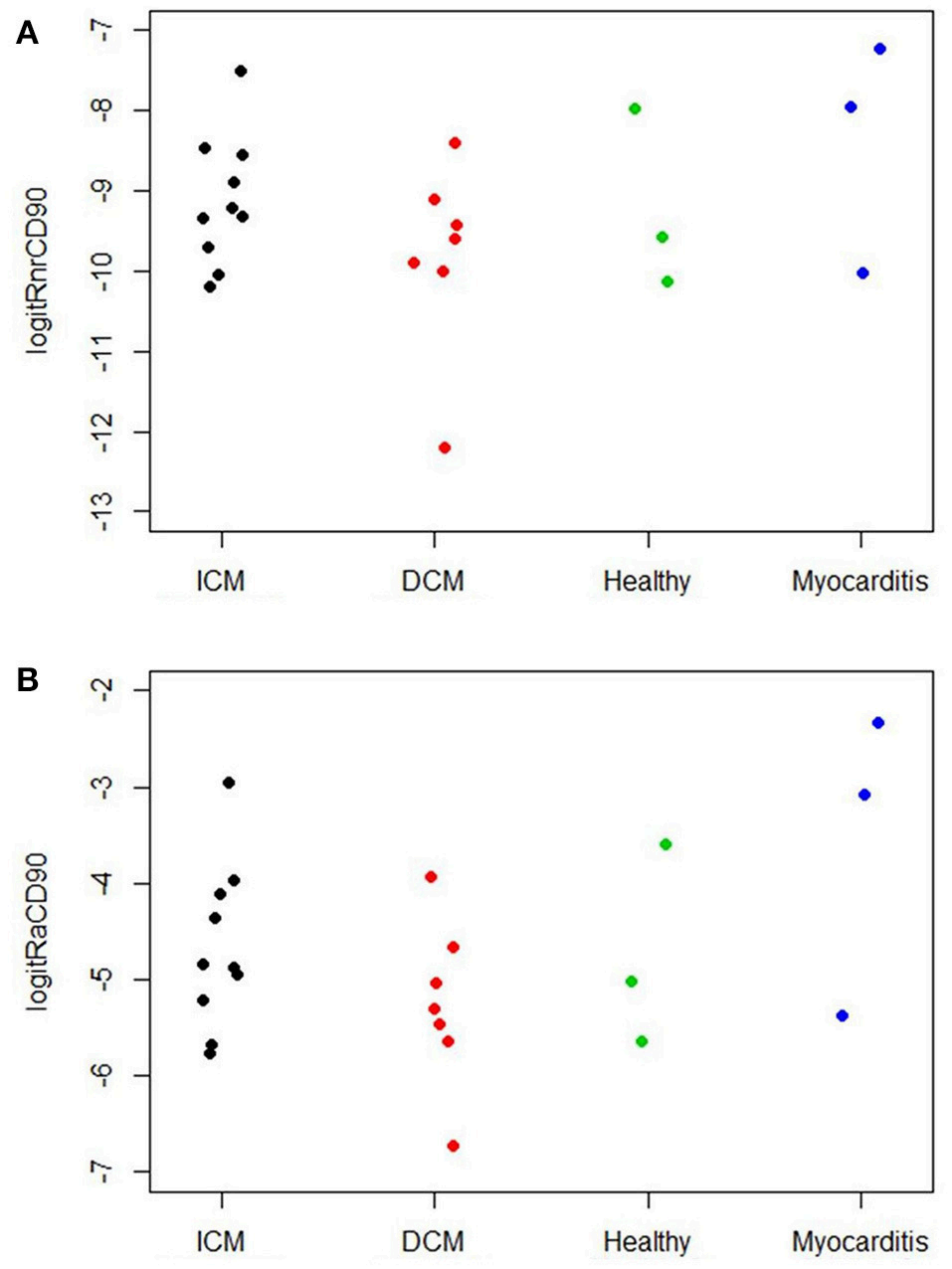
**(A)** Relative *number* of CD90^+^ cells per tissue section (*n* = 23). The X axis designates the patient groups: ICM (*n* = 10), DCM (*n* = 7), Healthy (*n* = 3), and Myocarditis (*n* = 3). The Y axis presents the relative number of CD90^+^ signals (total number of signals divided by total area of tissue section; Table 2), converted to the logit scale [logit(*p*) = log (*p*/(1 – *p*))]. Individual values of the patients are presented as spots. **(B)** Relative *area* of CD90 per tissue section (*n* = 23). The X axis designates the patient groups: ICM (*n* = 10), DCM (*n* = 7), Healthy (*n* = 3), and Myocarditis (*n* = 3). The Y axis presents the relative area of CD90^+^ signals (total area of signals divided by total area of tissue section; Table 2), converted to logit scale [logit(*p*) = log (*p*/(1 – *p*))]. Individual values are presented as spots.

The digital image analysis facilitated a specialized quantitative analysis of the whole slide to CD90^+^ and CD117^+^ signals. The relative *number* of CD90^+^ signals (total number of signals divided by the total area of tissue section) and the relative *area* of CD90^+^ signals (total area of signals divided by the total area of the tissue section) were calculated (Table 2). On comparison of the acute and chronic disease states of the heart in this study, it is striking to see that the highest expression of CD90^+^ cells was detected in the myocarditis group (Figure 11A). ICM and DCM groups have similar numbers of CD90^+^ cells or even lower, in comparison to healthy cardiac patients. The relative area of CD90 (Figure 11B) correlates with the relative number of CD90^+^ signals. The greatest relative area of CD90 was found in the myocarditis group, even though no statistical evidence for group differences was detected.

**Table 2:** Detailed patient information including biopsy location, diagnosis, and results of the digital image analysis (*n* = 23).

**Patient ID**	**Biopsy location**	**Diagnosis**	**Total number of CD90^+^ signals per tissue section**	**Relative number of CD90^+^ signals per tissue section**	**Relative area of CD90^+^ signals per tissue section**	**Relative area of CD117^+^ signals per tissue section**
Patient 1	Right ventricle	Healthy	1,766	0.00006964	0.00657148	0.15489015
Patient 2	Ventricle	Healthy	986	0.00034775	0.02691154	0.00128155
Patient 3	Ventricle	Healthy	357	0.00003978	0.00351804	0.02040115
Average			1,036	0.00015239	0.012333687	0.058857617
Patient 4	Septum	Myocarditis	14,493	0.00073061	0.08861789	0.28542232
Patient 5	Septum	Myocarditis	870	0.00004446	0.00457649	0.60600118
Patient 6	Septum	Myocarditis	13,860	0.00035446	0.04414038	0.64796254
Average			9,741	0.00037651	0.045778253	0.51312868
Patient 7	Septum	ICM	3,003	0.00009935	0.00788957	0.07027659
Patient 8	Left ventricle	ICM	7,000	0.00021093	0.01858989	0.01535276
Patient 9	Left ventricle	ICM	1,562	0.00008959	0.00703351	0.41275665
Patient 10	Septum	ICM	3,566	0.00013633	0.01256228	0.40487681
Patietn 11	Septum	ICM	1,230	0.00006138	0.00538673	0.48448345
Patient 12	Septum	ICM	12,254	0.00055332	0.04938569	0.13488475
Patient 13	Left ventricle	ICM	1,977	0.00008794	0.00760771	0.48799854
Patient 14	Left ventricle	ICM	1,190	0.0001929	0.01611067	0.57780017
Patient 15	Left ventricle	ICM	781	0.00003774	0.00313921	0.41058415
Patient 16	Left ventricle	ICM	793	0.00004349	0.00341586	0.32906417
Average			3,336	0.000151297	0.013112112	0.332807804
Patient 17	Left ventricle	DCM	1,634	0.00005087	0.00423627	0.15127794
Patient 18	Septum	DCM	626	0.00004535	0.00356114	0.21075096
Patient 19	Septum	DCM	4,203	0.00022521	0.01916202	0.45260291
Patient 20	Left ventricle	DCM	1,388	0.00006808	0.00492588	0.3632407
Patient 21	Septum	DCM	96	0.00000503	0.00120728	0.36082968
Patient 22	Left ventricle	DCM	3,251	0.00008052	0.00644762	0.1610318
Patient 23	Septum	DCM	3,808	0.00011138	0.00938711	0.30622664
Average			2,144	8.37771E-05	0.006989617	0.286565804

**Figure 12:**
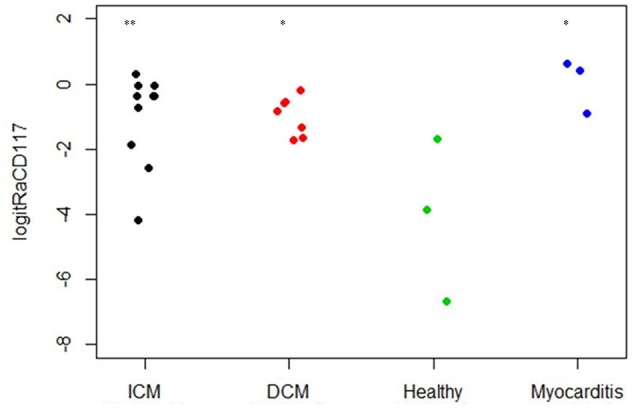
Relative *area* of CD117 per tissue section (*n* = 23). The X axis designates the patient groups: ICM (*n* = 10), DCM (*n* = 7), Healthy (*n* = 3), and Myocarditis (*n* = 3). The Y axis presents the relative area of CD117^+^ signals (total area of signals divided by total area of tissue section; Table 2), converted to logit scale [logit(*p*) = log (*p*/(1 – *p*))]. Individual values are presented as spots. The expression of CD117 is significantly higher in ICM, DCM and Myocarditis, compared to healthy (^*^P < 0.05 vs. healthy, ^**^P < 0.01 vs. healthy).

As CD117 staining included not only individual signals but also larger stained areas, the focus was set on the relative area of CD117^+^ signals (total area of signals divided by the total area of the tissue section) and not on the number of CD117^+^ signals (Table 2). The relative area of CD117^+^ signals is significantly increased in all three disease states: myocarditis, ICM and DCM, compared to healthy patients (*p* < 0.05; Figure 12).

### Co-expression of CD90 and CD117

**Figure 13:**
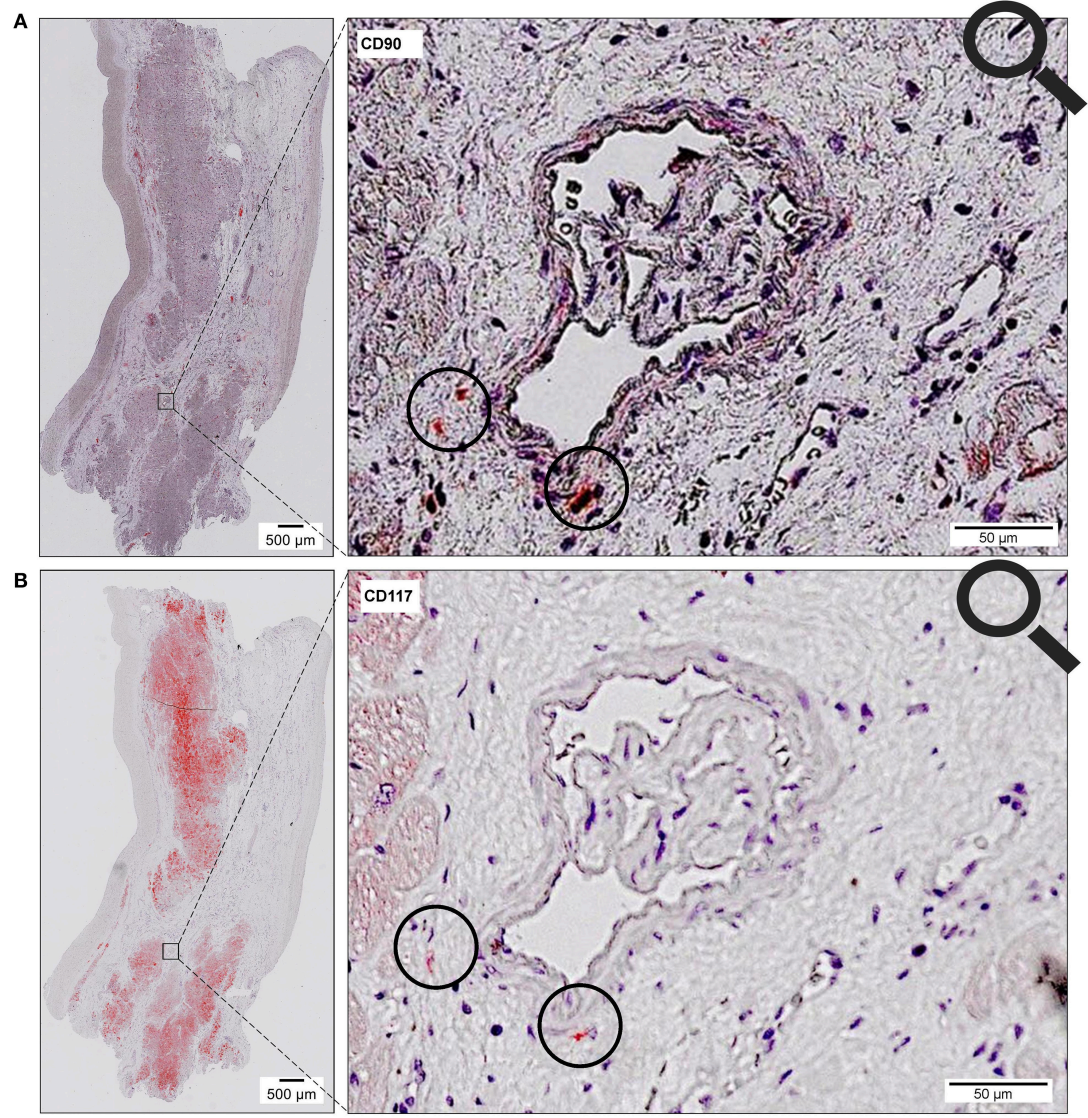
Coexpression of CD90 and CD117. Two serial sections of the same patient (Patient 4) are stained with different antibodies (**A**, CD90; **B**, CD117). The circle shows cells with co-expression of both stem cell markers (CD90^+^ on 1st slide, CD117^+^ on 2nd slide).

**Figure 14:**
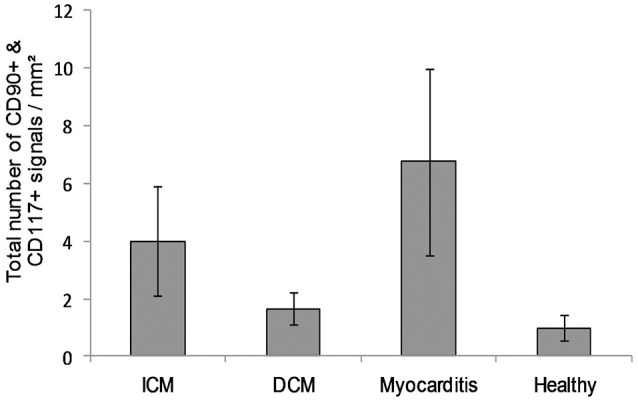
A number of cells with co-expression of CD90 and CD117 per square millimeter (*n* = 12). The data are expressed as mean ± standard deviation. The Myocarditis patient group displayed the most cells with co-expression of both stem cell markers.

**Figure 15:**
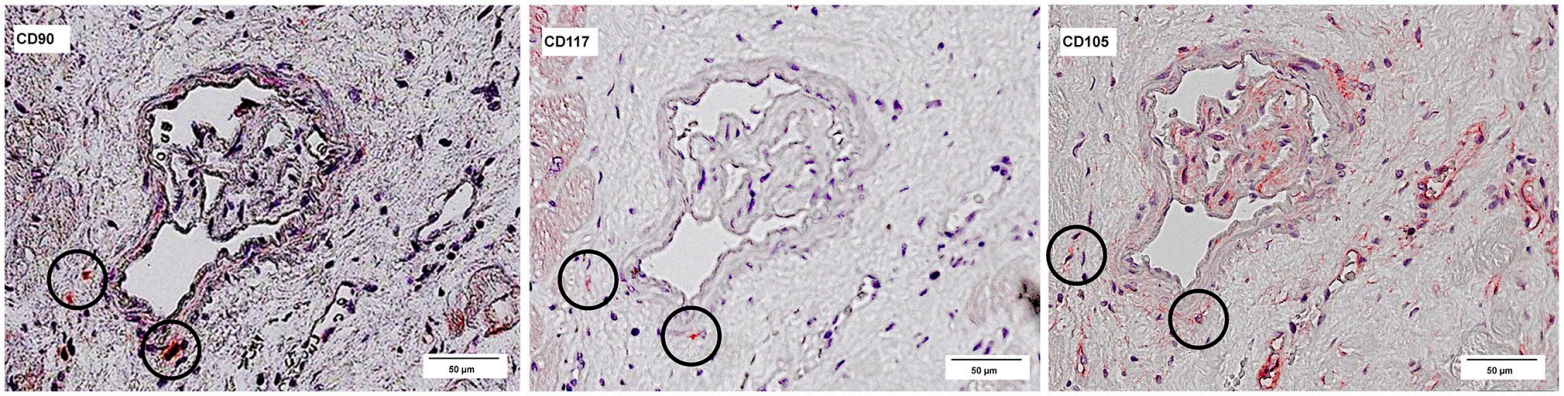
Co-expression of CD90, CD117, and CD105. Three serial sections of the same patient (Patient 4) are stained with different antibodies: CD90, CD117, and CD105. The circles mark cells, which are positive for all three antibodies.

The co-localized signals were evaluated and counted manually, based on the comparison of the histological images. CD90^+^ cells, which were co-localized with CD117, were identified in all patient groups (Figure 13). The lowest number of cells with co-expression was found in the group of cardiac healthy (1 ± 0.43 CD90^+^CD117^+^ cells/mm^2^), followed by DCM group (1.67 ± 0.58 cells/mm^2^) and patients with ICM (4 ± 1.89 cells/mm^2^; Figure 14). Patients with myocarditis had by far the highest number of cells with co-expression (6.75 ± 3.25 cells/mm^2^). These results also support our previous described outcome of an increase of CD90 and CD117, especially in myocarditis. The CD117^+^ signal directly in the cardiomyocytes was not detected in the CD90 stained sections. We conclude that these cells do not belong to the local progenitor cells. Furthermore, on some selected tissue samples we analyzed a third stem cell marker, CD105, and we identified cells with expression of all three stem cell markers (Figure 15).

## Discussion

Numerous stem cell markers were analyzed especially referring to CPCs. In the present study, the focus was mainly set on the co-expression of CD90 and CD117 in the human heart, which was already demonstrated (Gambini et al., 2011). In general, the CD90 marker is considered as a fibroblast marker but is also known as a mesenchymal stem cell (MSC) marker. Cardiac fibroblast research has been tremendously accelerated in the last decade (Gourdie et al., 2016). The novel therapeutic strategies in heart diseases with special focus on fibroblasts are reviewed elsewhere (Gourdie et al., 2016). In our previous studies, we have shown the multilineage potential of skin-derived CD90^+^ cells (Lorenz et al., 2008). Moreover, we have successfully treated acute and chronic diabetic wounds and skin ulcer of human cases by activation of local progenitor cells (Bader et al., 2011; Günter et al., 2013). In our opinion, it is important to note that the CD90 marker characterizes seemingly opposing states of cells; on the one hand fibroblasts involved in scar formation, but on the other hand, stem or progenitor cells, which could induce functional tissue regeneration. We believe that those CD90^+^ progenitor cells reside in local organs and are activated after injury. Previously, we have studied CD90 progenitor cells of the skin and shown their dependence toward localized cytokine stimulation for proliferation (Lorenz et al., 2008) and we proved the proliferation of local CD90^+^ progenitor cells after injury in the liver (unpublished data). Acute myocarditis is a process that is characterized by intense local inflammation as well. Even though the myocarditis group in the present experiments was very small (*n* = 3) due to the limited availability of the samples, the high expression of CD90^+^ cells corresponds to the fact that myocarditis patients more frequently undergo a complete healing pattern. The progenitor cell activation in myocarditis may explain the better functional outcome with less or no scar presence allowing the localized functional regeneration of cardiac tissue. In contrast, chronic disease states showed a depletion of CD90^+^ cells, correlating clinically to the unsuccessful healing outcomes.

Moreover, various researchers identified CD117^+^ cells in human myometrium (Ciontea et al., 2005), human fallopian tube (Popescu et al., 2005), human mammary gland stroma (Radu et al., 2005) as well as in human atrial myocardium (Hinescu et al., 2006), and ventricular myocardium (Popescu et al., 2006). We have proved here, that the number of CD117^+^ cells is upregulated in the diseased heart, especially in myocarditis. Our results validate the findings of previous studies; the number of CD117^+^ cells is increased in several cardiac diseases, for instance in advanced heart failure and aortic stenosis (Urbanek et al., 2003; Kubo et al., 2008; Itzhaki-Alfia et al., 2009). The human heart possesses a cardiac stem cell pool (Urbanek et al., 2005). The activation of the local stem cells occurs in response to ischemic injury. The stem cell pool has a crucial role in the regeneration of infarction heart (Urbanek et al., 2005). Matuszczak et al. reported that there are no differences in the number of CD117^+^ cells between various disease groups (Matuszczak et al., 2014). However, they had not used control tissue from healthy patients.

CD117^+^ local CPCs had already been used for the treatment of heart diseases in both human cases and animal models (Bolli et al., 2011). The method involves the isolation of autologous CD117^+^ cells, expansion *in vitro* and injection of those in high numbers (Bolli et al., 2011).

In comparison with conventional stem cell therapy, the activation of local endogenous progenitor cells is a holistic approach to both preventing and regulating the heart. We are aware that the characterization of progenitor cells requires the identification of various stem cell markers and it is of high importance to exclude unspecific signals. Here, we analyzed the co-expression of CD117, CD90, and partially CD105, also. Previous studies underline our analyses: Gambini and colleagues demonstrated a co-expression of CD117 and CD90 or rather CD105 in human heart auricle primary cultured cells (Gambini et al., 2011). Several other studies also identified CD117^+^ cells which were positive for CD105 and CD90, too (Li et al., 2012; Matuszczak et al., 2014). CD90 and CD105 were detected on cardiosphere-derived cells (CDCs) (Smith et al., 2007; Davis et al., 2009; Mishra et al., 2011; Chan et al., 2012). A series of other markers was proven on CD117^+^ cells, including CD29, CD44, CD31, CD34, and Sca1 (Gambini et al., 2011; Fang et al., 2012; Matuszczak et al., 2014). Furthermore, it was already displayed that CD117+ cardiac stem cells are negative for CD45 (Matuszczak et al., 2014) and that not all CD117^+^ cells are mast cells (Kubo et al., 2008; Zhou et al., 2010).

Vicinanza et al. also showed that cardiac CD117^+^/CD45^−^ cells are clonogenic and multipotent, but they determined that >90% of cardiac CD117^+^ cells contain endothelial cells and their precursors (Vicinanza et al., 2017). They demonstrated the myogenic and regenerative potential of CD117^+^/CD45^−^ cells in the damaged myocardium after injection of CD117^+^/CD45^−^ cells into damaged myocardium. Therefore, they conclude that CD117 is still an essential marker for CPCs (Vicinanza et al., 2017).

Nevertheless, future experiments with additional stem cell markers are necessary to prove the progenitor cell identity of CD117^+^ cardiac cells and to exclude unspecific labeling. We identified many CD117^+^ stained cardiomyocytes, which do not belong to the local progenitor cells. Other researchers studied CD117^+^ hematopoietic bone marrow cells and their ability to act as cardiac progenitors and to transdifferentiate into cardiomyocytes (Orlic et al., 2001; Rota et al., 2007). Rota et al. reported that CD117^+^ bone marrow cells lose their hematopoietic CD45 phenotype and obtain a cardiomyocyte phenotype (Rota et al., 2007). Probably these cells were also enriched in cases of inflammation or other pathological conditions. Further studies would be necessary to analyze this kind of cells.

In the present study, it was not our purpose to perform a series analysis of cardiac stem cell markers. Rather than that, the present study mainly focuses on investigating the combination of a traditional histological method with a novel digital image analysis technology. This technology enables a quantitative evaluation of two CPC markers in comparison of paraffin–embedded tissue sections of healthy and diseases heart samples. It would be of high interest to complement these experiments with other techniques such as immunofluorescence and high magnification confocal microscopy in the future.

In the present experiment, we identified CD90^+^ and CD117^+^ cells in all patient groups: Myocarditis, ICM, DCM, and healthy cardiac patients. With the novel digital image analysis technology, a comparison of differently sized paraffin-embedded tissue sections is available. Taking into consideration that the sample size in the present experiment was limited, we proved an increase of CD90^+^/CD117^+^ cells in acute myocarditis. This finding supports our theory that endogenous cardiac stem or progenitor cell activation is part of the repairing mechanism after acute myocardial damage, as in cases of acute myocarditis. A similar inflammation process is described for acute myocardial infarction. Nevertheless, in most cases, acute myocardial infarction leads to the development of a scar. Further studies may show, how an amplification of the local myocardial stem or progenitor cell activation may contribute to the myocardial regeneration and healing process.

## Conclusion

This study aimed at the identification and the quantitative analysis of CD90^+^ and CD117^+^ cardiac cells from human myocardium biopsies of 23 patients. Besides the conventional histological image analysis, the digital image analysis enabled a computer-based immunohistochemical quantification of some stem cell markers, using whole-slide images created by the virtual slide scanning microscopy. In our experiments, the number of CD90 and CD117 signals in patients with myocarditis was higher than in all other groups. Taking into consideration the regenerative healing potential prospects of myocarditis, it is likely that there is a relation between CD90 and CD117 expression and clinical outcome. Future studies with larger sample size are necessary to confirm that theory. The proof on the existence of endogenous resident progenitor cells not only in the healthy but also in the diseased human heart opens up the promising concept of regeneration instead of repair, which has an impressive scope in treating or preventing cardiovascular diseases.

## Author contributions

MG, JS, SE, SG, and AB: conceived and designed the experiments; NS, WM, and JS: organized the human tissue samples; MG and SE: performed the experiments; MG, SE, BB-S, and MA: analyzed the data; MG, JS, SE, SG, AB, and MA: wrote the paper; SG and AB: provided guidance on the whole study.

### Conflict of interest statement

The authors declare that the research was conducted in the absence of any commercial or financial relationships that could be construed as a potential conflict of interest. Maria Athelogou was employed by company Definiens AG, Munich, Germany. All other authors declare no competing interests.
