# Supplementary material for: Qualitative and Quantitative Analysis of Cardiac Progenitor Cells in Cases of Myocarditis and Cardiomyopathy
Source: Front Genet. 2018 Mar 6;9:72. doi: 10.3389/fgene.2018.00072 (PMC5845648; doi:10.3389/fgene.2018.00072)
Supplement: Supplementary file 1 [file DataSheet1.pdf]

Positive, negative and IgG - controls for Immunohistochemistry (IHC)

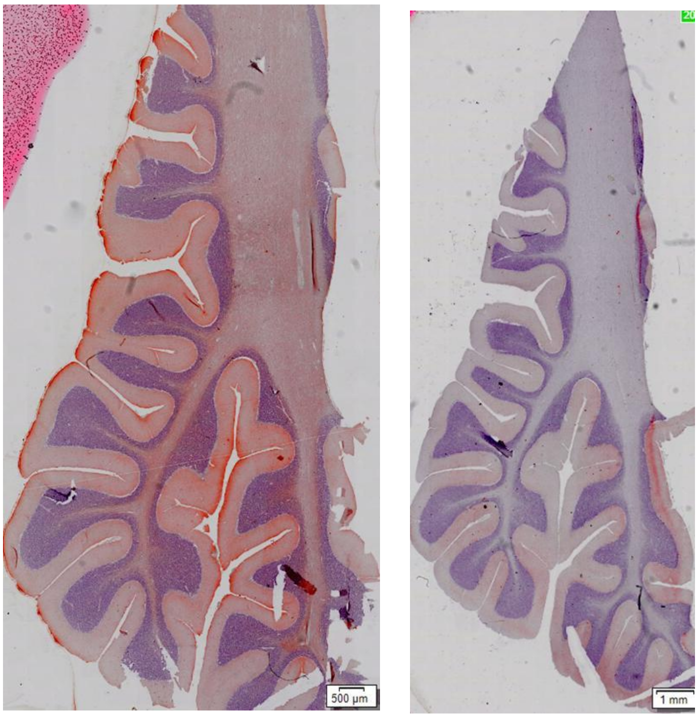

A **B**

Supplementary Figure 1. Histological images of the positive controls (human cerebellum, **A**: CD90 and **B**: CD117)

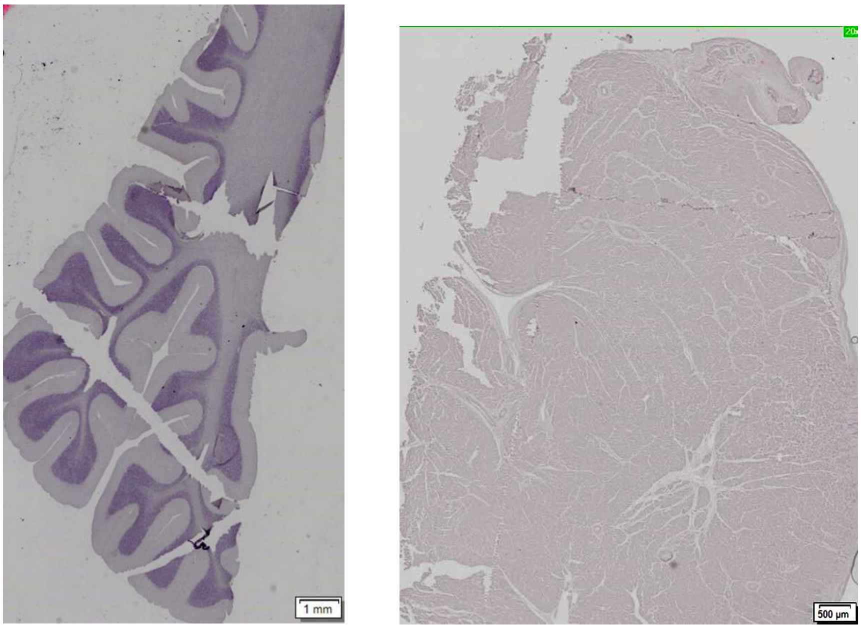

C **D**

Supplementary Figure 2. Histological images of the IgG – control (**C:** human cerebellum, **D**: human myocardium)

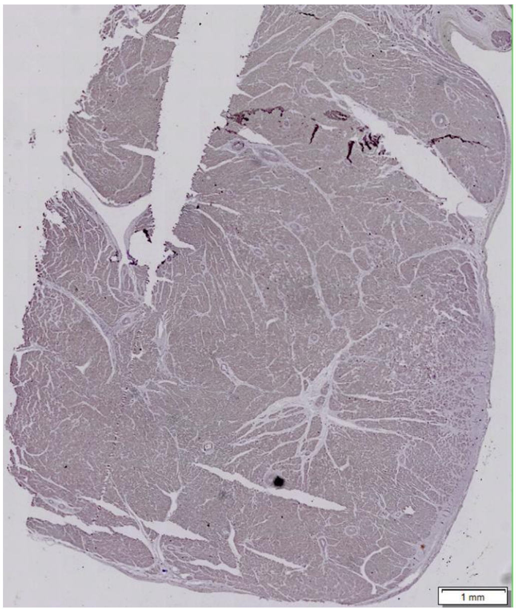

E

Supplementary Figure 3. Histological image of the negative control (**E**: human myocardium)
